# Effective transfer of tumor annotations from hematoxylin and eosin to fluorescence images of breast and lung tissues

**DOI:** 10.1117/1.JBO.31.1.016501

**Published:** 2025-12-24

**Authors:** Tianling Niu, Emi Ampo, Julie M. Jorns, Mollie Patton, Tongtong Lu, Dong Hye Ye, Tina W. F. Yen, Bing Yu

**Affiliations:** a Marquette University and Medical College of Wisconsin, Joint Department of Biomedical Engineering, Milwaukee, Wisconsin, United States; b Medical College of Wisconsin, Department of Surgery, Milwaukee, Wisconsin, United States; c Medical College of Wisconsin, Department of Pathology and Laboratory Medicine, Milwaukee, Wisconsin, United States; d University of Wisconsin Oshkosh, Department of Engineering and Engineering Technology, Oshkosh, Wisconsin, United States; e Georgia State University, Department of Computer Science, Atlanta, Georgia, United States

**Keywords:** fluorescence imaging, microscopy with ultraviolet surface excitation, annotation transfer, intraoperative assessment

## Abstract

**Significance:**

Accurate transfer of annotations from histological images to fluorescence images is essential in developing deep learning (DL)-based optical imaging systems for intraoperative assessment of tumor margins. Manual annotation is time-consuming, prone to interobserver variability, and impractical for large-scale datasets.

**Aim:**

We present a semi-automated method that can effectively transfer tumor annotations from pathologist-annotated hematoxylin and eosin (H&E) images to fluorescence images captured using microscopy with ultraviolet surface excitation (MUSE). This method is not intended for intraoperative use but rather to facilitate the creation of annotated datasets for DL model development.

**Approach:**

Our semi-automated method consists of nonrigid image registration, outline extraction and refinement, and annotation transfer. The method was applied to H&E and MUSE image pairs from 35 breast and lung tissue samples. Manual annotations in MUSE images were used as the ground truth for evaluation.

**Results:**

The proposed method achieved a Dice score coefficient of 0.87±0.07, convolutional-neural-network-based feature similarity of 0.94±0.04, and a normalized Hausdorff distance of 0.15±0.06 across the dataset.

**Conclusion:**

These results demonstrate that the method provides a fast and accurate solution for generating annotated MUSE datasets necessary for training DL algorithms for intraoperative tumor margin detection.

## 1 Introduction

Breast and lung cancers rank among the most prevalent and lethal malignancies worldwide, necessitating improved diagnostic and treatment strategies. In the United States, breast cancer is the most common malignancy and remains one of the leading causes of cancer-related mortality in women, with over 310,720 new cases and ∼42,250 deaths projected for 2024.^1^ Lung cancer, the leading cause of cancer-related mortality, accounts for more than 127,000 deaths annually, underscoring its critical public health burden.^1^ Surgical resection plays an important role in the treatment of patients with resectable breast and lung cancer.^2^^,^^3^ Complete tumor removal while preserving healthy tissue is essential in both breast-conserving surgery and lung tumor resection to reduce the likelihood of local cancer recurrence, which translates to improved survival rates.^2^^,^^4^^,^^5^ This hinges on effective intraoperative margin assessment (IMA), which remains challenging with existing technologies, including frozen section analysis, touch preparation cytology, and the MarginProbe device.^6^^–^^8^ Particularly, frozen section and touch preparation require significant resources and specialized expertise,^9^^,^^10^ whereas the MarginProbe, despite its ease of use, has high false positive rates in clinical studies.^11^ These limitations underscore the need for improved IMA methods that are both effective and accessible.

Emerging technologies, such as mass spectrometry imaging (MSI),^12^^,^^13^ total internal reflection fluorescence (TIRF) microscopy,^14^ confocal microscopy,^15^^–^^17^ spatial frequency domain imaging,^18^^,^^19^ wide-field fluorescence imaging, light sheet microscopy,^20^ impedance spectroscopy,^21^^,^^22^ optical coherence tomography (OCT),^23^^,^^24^ and microscopy with ultraviolet surface excitation (MUSE),^25^^–^^27^ have addressed or have the potential to address these limitations. Among these emerging technologies, fluorescence-based wide-field, confocal, light sheet, and MUSE microscopy offer a promising solution by delivering high-resolution images of cellular and tissue structures from unprocessed tissue surfaces, bypassing the time and labor associated with traditional methods.^25^^–^^27^ Among them, MUSE uses deep ultraviolet (DUV) light to excite native tissue fluorophores, such as nicotinamide adenine dinucleotide (NADH), collagen, and elastin, or extrinsic fluorophores, such as Hoechst, propidium iodide, eosin Y, and rhodamine, which emit distinct fluorescence signals based on their biochemical properties. MUSE allows generating images of the surfaces of large tissue areas with resolution comparable to conventional histology within minutes, ideal for intraoperative assessment of positive tumor margins. Initial studies have shown that MUSE can accurately identify cancerous tissues and delineate tumor margins, thus having the potential to enhance the surgeon’s ability to achieve complete tumor resection and reduce the risk of recurrence.^26^^,^^28^^–^^31^ Moreover, MUSE has shown efficacy across a range of cancers, including breast, head and neck, lung, and skin cancers, where real-time margin assessment is critical.^25^^,^^26^^,^^28^^–^^31^ As MUSE technology continues to advance, it holds the potential to become an invaluable tool in surgical oncology, providing both the diagnostic accuracy and speed essential for improving patient outcomes.

Deep learning (DL) has become increasingly used in analyzing fluorescence images for cancer diagnosis, providing enhanced precision in identifying cancer cells and differentiating them from healthy tissue.^29^^,^^30^^,^^32^^–^^36^ By leveraging convolutional neural networks (CNNs) and other advanced architectures, DL models can process complex, high-dimensional data from fluorescence images, allowing for the detection of subtle patterns and biomarkers of cancer. This automated, data-driven approach improves upon traditional, often manual image analysis methods by reducing observer variability and increasing diagnostic accuracy. Furthermore, DL enables the rapid and efficient analysis of vast datasets, potentially accelerating diagnosis and enabling personalized treatment plans based on detailed cellular and molecular profiles.^37^ As a result, DL technologies are rapidly being adopted in both research and clinical settings, offering promise for earlier and more reliable cancer detection. DL-based cancer diagnosis, however, relies heavily on large datasets of annotated training and test images with well-defined ground truth to achieve high diagnostic accuracy.^37^^–^^42^ Annotated images, labeled by expert pathologists, are essential for training DL algorithms to recognize patterns associated with cancerous and healthy tissues, as well as specific tissue subtypes. Large, diverse datasets along with ground truth labels help DL models generalize well to different cases by capturing a wide range of tissue variations, staining methods, and imaging artifacts, which is crucial for real-world applications.^40^^,^^43^ Moreover, a substantial number of annotated test images are needed to thoroughly evaluate the model’s performance, ensuring it accurately detects and classifies cancer with minimal false positives and negatives. Consequently, building these large, annotated image datasets is a crucial step for advancing DL models in surgical oncology.

Development of an effective DL-based IMA tool using fluorescence imaging technologies such as MUSE also relies heavily on annotated datasets as the ground truth. However, pathologists are primarily trained to interpret hematoxylin and eosin (H&E) stained images, which are widely regarded as the gold standard in histopathological diagnosis. By contrast, MUSE images are less familiar to pathologists. On the other hand, there has been a national shortage of pathologists since 2007,^44^ making it more difficult for researchers to rely on a pathologist to annotate fluorescence images. Therefore, using annotations from H&E images as the reference standard and transferring them to corresponding MUSE images is essential for ensuring diagnostic relevance and consistency. Commonly used manual annotation of hundreds or even thousands of fluorescence tissue images is not only tedious, slow, and costly, but also faces many hurdles, particularly for MUSE images, which often require trained experts to be familiar with their unique appearance and contrast. First, DUV light has a penetration depth in biological tissue limited to ∼10  μm,^45^^,^^46^ but the cutting depth from the top surface of the tissue block during the formalin-fixed, paraffin-embedded (FFPE) process varies from 0 to 200  μm, complicating direct image registration. Second, a co-registration process needs to accommodate various deformations, such as tissue losses, folding, stretching, shrinking, or warping, that each tissue sample might undergo during the FFPE process.^47^ Third, compared with H&E images, MUSE and many other fluorescence images are collected with lower magnification and different fluorophores, resulting in substantial difference in contrasts, appearance, and resolution. Consequently, transferring outlines of tissue subtypes and structures from H&E to MUSE images is challenging for both manual and computer-based methods. Several commercial software, such as Nikon NIS (semi-automated), AutoAligner by Bitplane Scientific (manual and automatic), TurboReg (a plugin for ImageJ), and open source software like MMIR^48^ are available for co-registration of multimodality images, but most of these tools have been primarily developed for registering small image patches (1024×1024  pixels) between closely related modalities, and their use of grayscale images limits their direct applicability to MUSE images, which are high resolution and colored.^49^

Here, we report a new method that can be used to semi-automatically transfer outlines of tumor regions from pathologist-annotated H&E images to corresponding fluorescence images, streamlining the process of generating ground truth labeled data for the classification tasks using DL models. The tool takes advantage of recent advances in image processing to address the unmet need of the optical imaging society, particularly where various emerging fluorescence imaging technologies or new applications of such technologies need to be validated by pathological diagnosis as the gold standard. By overcoming challenges such as tissue deformation, contrast differences, and resolution disparities, our approach can accelerate the validation of emerging fluorescence imaging modalities. Compared with the manual annotation process, the tool is significantly faster, more accurate, and cost-effective, thus shortening the time required to develop annotated datasets for DL in emerging fluorescence imaging technologies.

## 2 Methodology

**Fig. 1:**
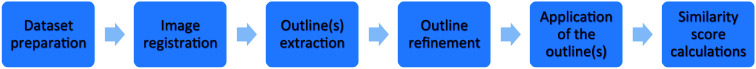
Workflow of the semi-automated annotation process.

The semi-automatic annotation method consists of multiple steps, including dataset preparation, image registration, outline(s) extraction from the pathologist-annotated H&E image, outline refinement, application of the outline(s) to the corresponding MUSE image, comparison annotations obtained using the semi-automated and manual method, and calculation of similarity scores between the two annotation sets, as illustrated in Fig. 1.

### 2.1 Dataset Preparation

**Fig. 2:**
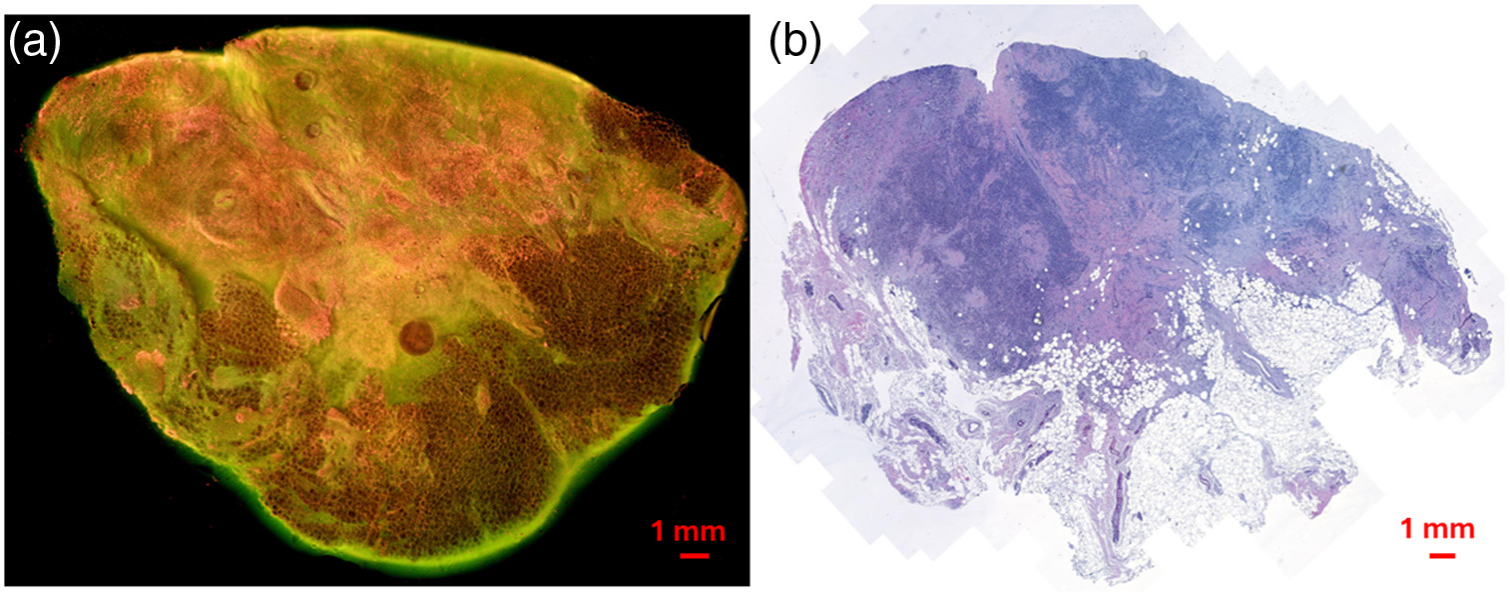
Screenshots of raw MUSE (a) and corresponding H&E (b) images from a breast sample, including both malignant and normal tissues (the presented images are at reduced resolution).

Tissue samples were obtained from the Medical College of Wisconsin Tissue Bank (MCW-TB). MUSE images were acquired using our MUSE setup in the lab.^27^ Freshly excised tissue samples were stained with propidium iodide, which stains cell nuclei, and eosin Y, which stains cytoplasm and connective tissue, to enhance image contrast.^27^ After staining, the samples were raster-scanned using the MUSE system to capture the fluorescence images. To ensure that the MUSE and H&E images correspond to the same tissue side, a marker was applied to the nonimaged face of each sample before returning it to the MCW-TB. This step guaranteed that the same face of the tissue block was consistently used for both MUSE and H&E imaging, preserving spatial correspondence. Following image acquisition, the tissue samples were returned to the MCW-TB for FFPE processing, H&E staining, and generation of corresponding histological images for tumor annotations. MUSE image blocks were stitched to obtain a whole sample image (WSI) in the JPEG format, whereas H&E images were scanned in the MIRAX format, as shown in Figs. 2(a) and 2(b), respectively. The study has collected a total of 35 pairs of H&E and MUSE images from breast or lung tissue samples that contain both malignant and normal tissues. The sample demographic information is summarized in Table 1. The H&E images were annotated for tumorous region(s) by a board-certified pathologist (Jorns) and shared with the research team. Manual annotations of the MUSE images were performed by a medical student (Ampo) based on the annotated H&E images, and the tumor outlines were confirmed by the pathologist.

**Table 1:** Summary of tissue demographic information.

Carcinoma type		Number of cases			
		Grade I	Grade II	Grade III	Total
**Breast tumor**	Invasive ductal carcinoma	3	11	1	15
	Invasive lobular carcinoma	5	6	0	11
	Invasive mucinous carcinoma	1	0	0	1
	Metastatic carcinoma (lymph node)	0	0	1	1
	Subtotal	9	17	2	28
**Lung tumor**	Adenocarcinoma	0	4	1	5
	Neuroendocrine carcinoma	0	0	1	1
	Metastatic leiomyosarcoma	0	1	0	1
	Subtotal	0	5	2	7
**Total**					**35**

### 2.2 Image Registration

Rigid or affine transformations such as translation, rotation, or scaling may not provide accurate alignment in registration between H&E and MUSE images due to their different resolutions and local deformation.^50^^,^^51^ Therefore, flexible, nonrigid, or elastic registration techniques were used to align the tissue correctly in MUSE and H&E images, even though there was no guarantee of consistency in the deformation metrics. The purpose of introducing nonrigid registration is to deform local areas of MUSE images to compensate for the stretching or missing area in H&E images. Global conventional rigid and affine registration approaches were not suitable for our samples and often resulted in lower performance as the H&E and MUSE images often exhibited different and unpredictable deformation metrics.^52^^,^^53^ Therefore, we employed a semi-automated method that uses manually selected paired points and applies a second-order polynomial transformation, allowing local nonlinear deformation of the MUSE images to compensate for distortions present in the H&E images.

There is no standardized or fixed deformation model for the registration process because each sample might have undergone unique stretching, shrinking, or warping during the FFPE process. More importantly, the cutting depth from the tissue block can also vary, meaning that sections may not be taken from the same depth, which further complicates direct image registration. Therefore, we introduced a semi-automated registration method that was based on manually selecting paired points in H&E and MUSE images. The registration metrics varied in tissue samples, allowing for more flexibility in addressing unpredictable deformations caused by the FFPE process and variations in cutting depth. At least six pairs of corresponding points were selected in both image modalities of the same tissue through visual inspection and recorded, with the points from the pathologist-annotated H&E image referred to as fixed points and those from the MUSE image as moving points. Selection of the points was an iteration process in which a Dice score coefficient (DSC) score was computed and maximized. This process has been repeated by three researchers (Niu, Ampo, and Lu) to mitigate potential errors introduced by manual point selection during registration. Selecting corresponding points along the tissue boundaries typically yields more accurate registration as these regions provide strong and distinct geometric features that are preserved across modalities.

**Fig. 3:**
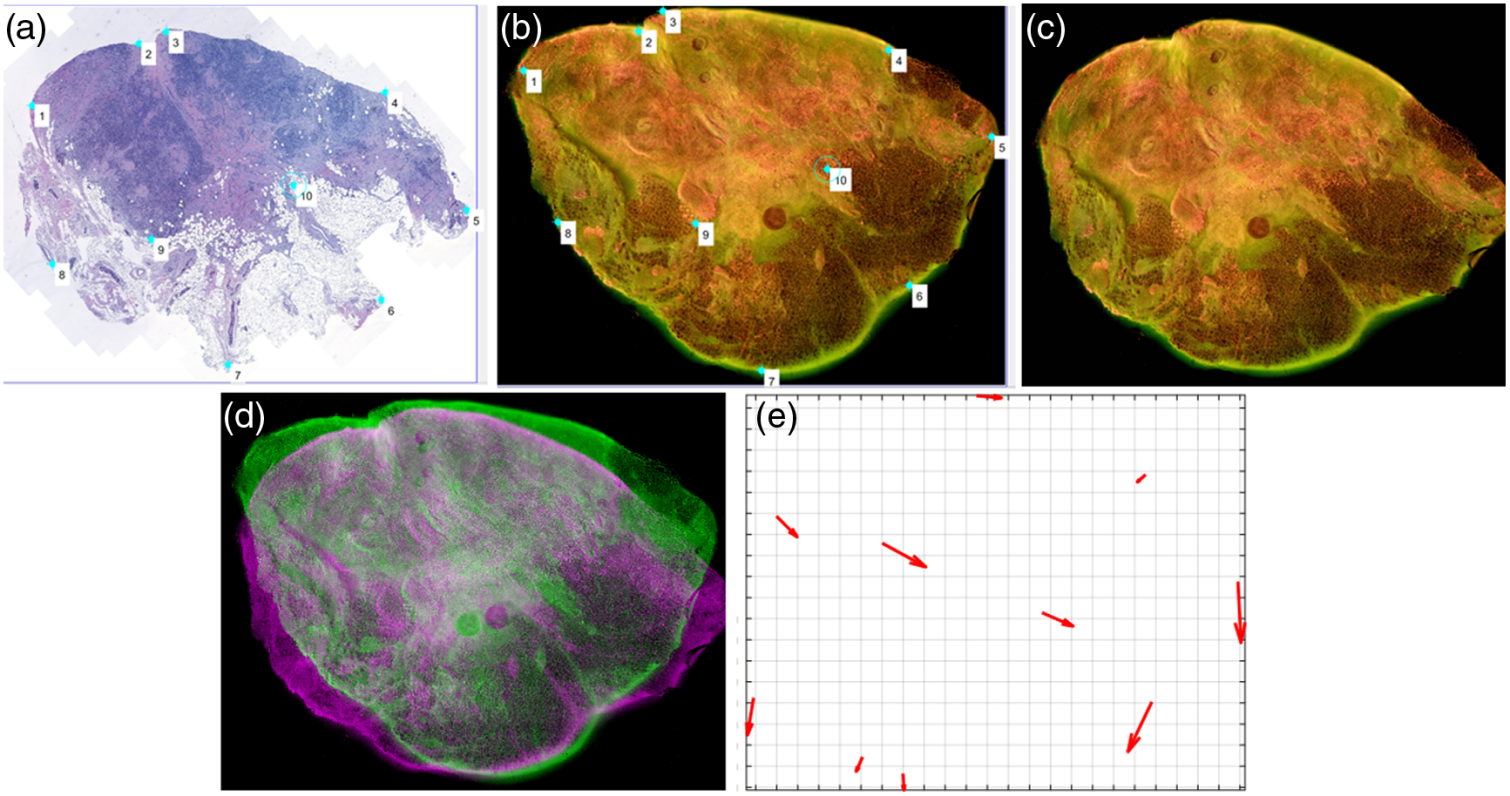
One example illustrating the image registration process. (a) manually selected points on H&E image; (b) manually selected paired points on MUSE image; (c) registered MUSE image based on the second-order polynomial transformation with coefficients $a_{0}=0.1681$, $a_{1}=0.9862$, $a_{2}=0.1141$, $a_{3}=0.0281$, $a_{4}=-0.0755$, $a_{5}=-0.1059$; $b_{0}=0.1227$, $b_{1}=-0.0214$, $b_{2}=0.9980$, $b_{3}=0.0635$, $b_{4}=-0.1174$, $b_{5}=0.0190$, obtained using the paired points; (d) the overlay comparison between the MUSE images before and after the registration; and (e) the registration metric.

Next, a second-order polynomial transformation was applied to register the MUSE image to the H&E image based on these paired points, accommodating the complex, nonlinear distortions in the tissue,^54^ defined as

$$X=a_{0}+a_{1}x+a_{2}y+a_{3}x^{2}+a_{4}xy+a_{5}y^{2},$$ (1)

$$Y=b_{0}+b_{1}x+b_{2}y+b_{3}x^{2}+b_{4}xy+b_{5}y^{2},$$ (2)

where (x,y) is the pixel position in the raw MUSE image, (X,Y) is the pixel position in the registered MUSE image, and a0,a1,…,a5 and b0,b1,…,b5 are coefficients calculated using the selected paired points. Figures 3(a) and 3(b) illustrate an example of the selected point pairs, and Fig. 3(c) displays the registered MUSE image. Figure 3(d) shows the tissue differences before and after registration, and Fig. 3(e) presents the corresponding registration metric. The registered points varied across tissue samples, reflecting the inherent variability in tissue deformation during the FFPE process. This second-order polynomial transformation allows flexible, nonlinear deformation while remaining computationally efficient and deforms the MUSE images based on the manually selected paired points.^52^^,^^53^^,^^55^ By doing so, it effectively compensates for local deformations present in the H&E images, enabling more accurate alignment of corresponding tissue structures and improving the reliability of annotation transfer.

### 2.3 Outline Extraction and Refinement

**Fig. 4:**
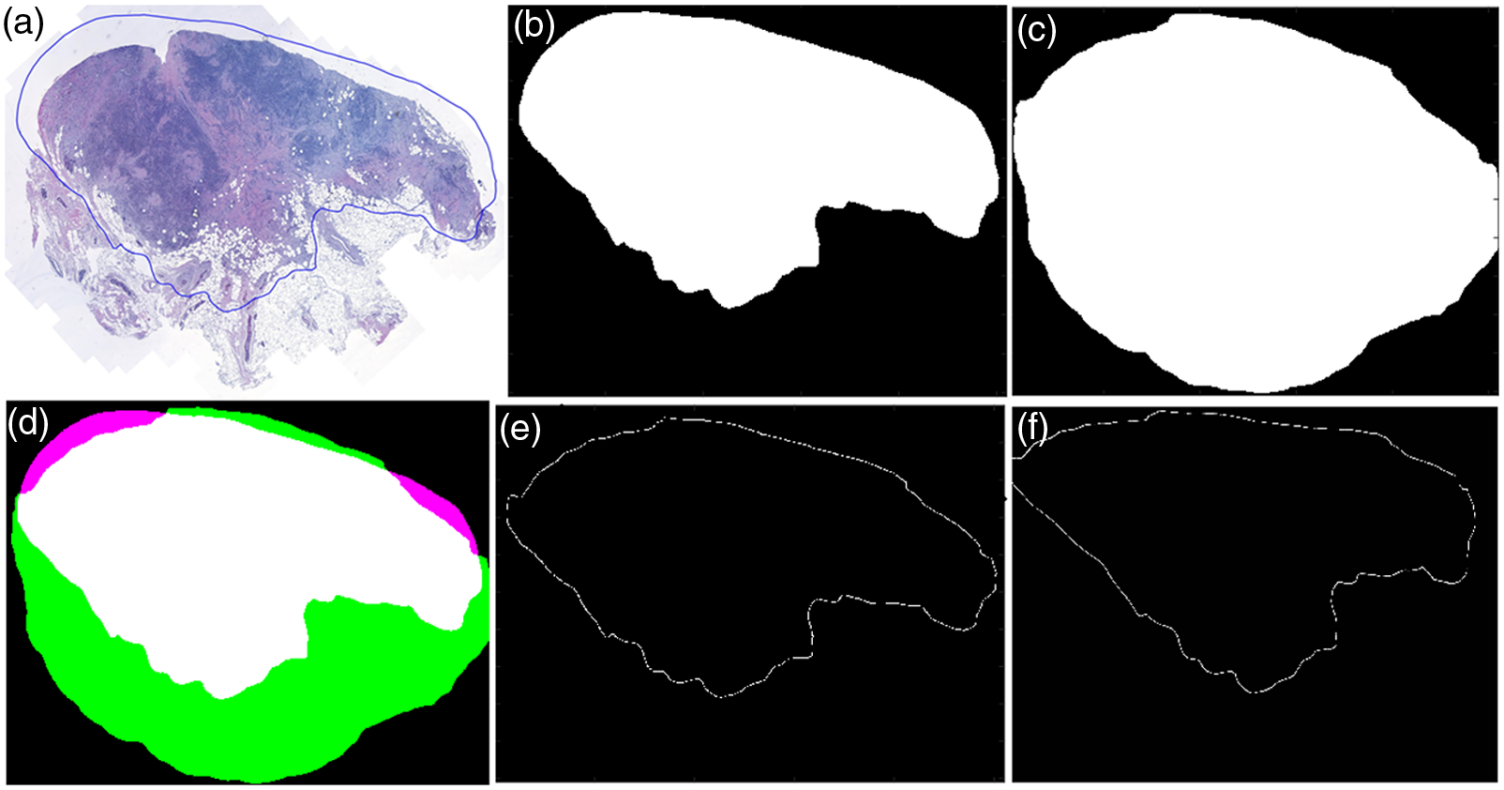
Example of annotation extraction and refinement. (a) Annotated H&E image; (b) extracted annotation area from panel (a); (c) tissue mask created based on [Fig. 3(c)] via Canny edge detection; (d) overlap between panels (b) and (c) (the white area is the overlap, and magenta and green areas are the differences); (e) annotation extracted from panel (d) border line of the white area; and (f) inverse registered annotation.

The tumor outline(s) from the pathologist-annotated H&E image was obtained by a simple subtraction arithmetic algorithm. Two files were exported from the H&E image in CaseViewer, a free slide viewing software, one with annotation from the pathologists and the other without. Next, the exact outline was obtained by subtracting the image without outline from the one with it. Because the outline was not always fully closed, we used the morphological structuring element to close and enhance it.^56^^,^^57^ This resulted in the first mask, named the annotation mask, as illustrated in Figs. 4(a) and 4(b).

The annotation outline extracted from the H&E images may include background regions based on pathologists’ experience as these areas could potentially contain tumor cells from deeper layers. This, however, was not applicable to MUSE images. Therefore, we performed the Canny edge detector^58^ in the registered MUSE images [Fig. 3(c)] in grayscale to separate tissue from background, generating a secondary mask referred to as the tissue mask. Gaps in the detected edges were closed using a disk-shaped structuring element (size 55) to produce an initial mask. A large Gaussian filter (size 63, σ=53) was then applied to smooth the mask, reducing noise and refining object boundaries. Finally, the object mask produced a clean binary mask by thresholding at 0.1 to move the dark background and highlighting the detected tissue regions, as shown in Fig. 4(c). The refined annotation was defined as the overlapping region (in white) between the initial annotation mask and the tissue mask, aiming to remove as much background as possible, as illustrated in Fig. 4(d). This refined annotation, shown in Fig. 4(e), was extracted using an erosion mask, which was generated by applying morphological erosion to the tissue mask to remove boundary artifacts and retain only the tumor region. This annotation was then inverse-registered, as shown in Fig. 4(f), and applied to the original MUSE image to obtain the final annotated MUSE image.

### 2.4 Calculation of Similarity Scores

**Fig. 5:**
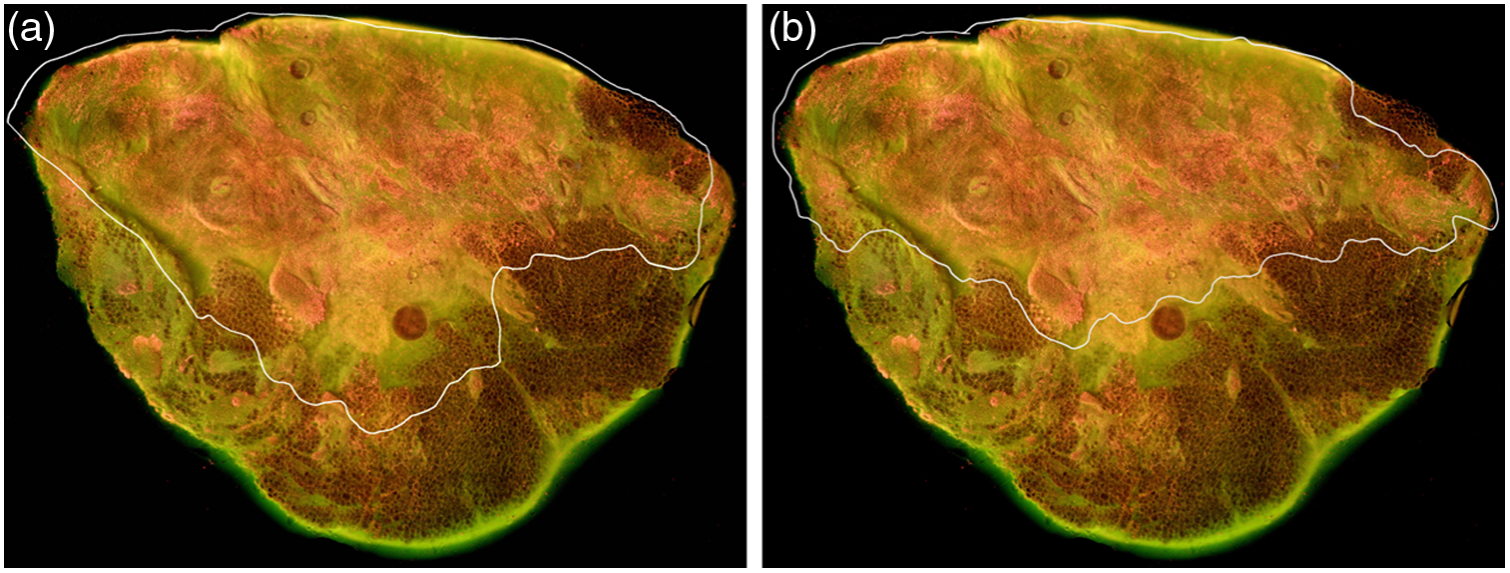
Annotated MUSE images. (a) Semi-automatically annotated image; (b) manually annotated raw image. The comparison was calculated between panels (a) and (b) with DSC = 0.883, CNN similarity = 0.981, and Hausdorff distance over picture size = 0.145.

The last step was to determine the accuracy of the semi-automatic method. Manual annotations on MUSE images were initially performed by a trained medical student (Ampo) and guided by the corresponding H&E annotations created by the board-certified pathologist Dr. Jorns and confirmed by pathology fellow Dr. Manjee. These manual MUSE annotations were subsequently reviewed and validated by Dr. Jorns to ensure accuracy and rigor. The resulting validated annotations were used as the gold standard to evaluate the performance of the semi-automated method. Three key metrics, including the DSC,^59^^–^^61^ CNN-based feature similarity,^62^ and Hausdorff distance^63^^,^^64^ were used to evaluate the accuracy of the semi-automatic method, as indicated in Fig. 5; Fig. 5(a) is the semi-annotated annotation, and Fig. 5(b) is the manual annotation. DSC quantifies the overall overlap between the semi-automated and manual annotations, which is a commonly used metric for annotation-related analysis. CNN-based feature similarity assesses the similarity of high-level features extracted between the annotated regions, providing a measure of structural consistency. Hausdorff distance evaluates the maximum boundary deviation between the manual and semi-automated annotations. Together, these metrics offer a comprehensive evaluation of both global and local accuracy, as well as structural fidelity, ensuring an objective assessment of the semi-automated method.

#### 2.4.1 DICE similarity coefficient

The DSC, defined as the ratio of the intersection of two contours to their union, was calculated using Eq. (3) to determine the similarity of the tumor outlines in the registered MUSE image pairs obtained using the two approaches (semi-automatic versus manual).

$$DSC=\frac{2\times |I_{1}\cap I_{2}|}{\left|I_{1}|+|I_{2}\right|},$$ (3)

where $I_{1}$ and $I_{2}$ denote binary images corresponding to the manual and semi-automated annotated MUSE images, respectively. A DSC score of 0 indicates no overlap, whereas a DSC score of 1 indicates a perfect overlap between the manually and semi-automatically obtained outlines.^59^^–^^61^

#### 2.4.2 CNN-based feature similarity

This metric calculates the cosine similarity between features extracted by a pretrained AlexNet CNN. AlexNet has been trained on over a million images and is capable of classifying images into 1000 object categories and allows them to compare annotations based on the high-level content while ignoring irrelevant factors such as scale and intensity variations. The network has learned rich feature representations from a diverse range of images, making it highly effective at capturing meaningful patterns. Here, it extracts high-level, fully connected features that represent abstract visual patterns in the MUSE image. This capability enables it to serve as an additional metric for evaluating the similarity between manual and semi-automatic tumor annotations.

#### 2.4.3 Hausdorff distance

Hausdorff distance, a measurement that calculates the maximum distance between two images, was employed to determine the maximum distance between the manual- and algorithm-generated annotations for the same sample.^63^^,^^64^ The Hausdorff distance between the two annotation lines was obtained by

$$H=[(x^{\prime },y^{\prime }),(X^{\prime },Y^{\prime })]$$ (4)

where $\left(x^{\prime },y^{\prime }\right)$ and $\left(X^{\prime },Y^{\prime }\right)$ represent the pixel positions of the annotation from the manual and semi-automated MUSE images, respectively. As the size of each MUSE image varies, comparisons were normalized by H over the maximum image dimension. A smaller percentage indicates a closer distance between manual and algorithm-generated outlines. The Hausdorff distance was used to evaluate the spatial difference between tumor annotations.

## 3 Results

**Fig. 6:**
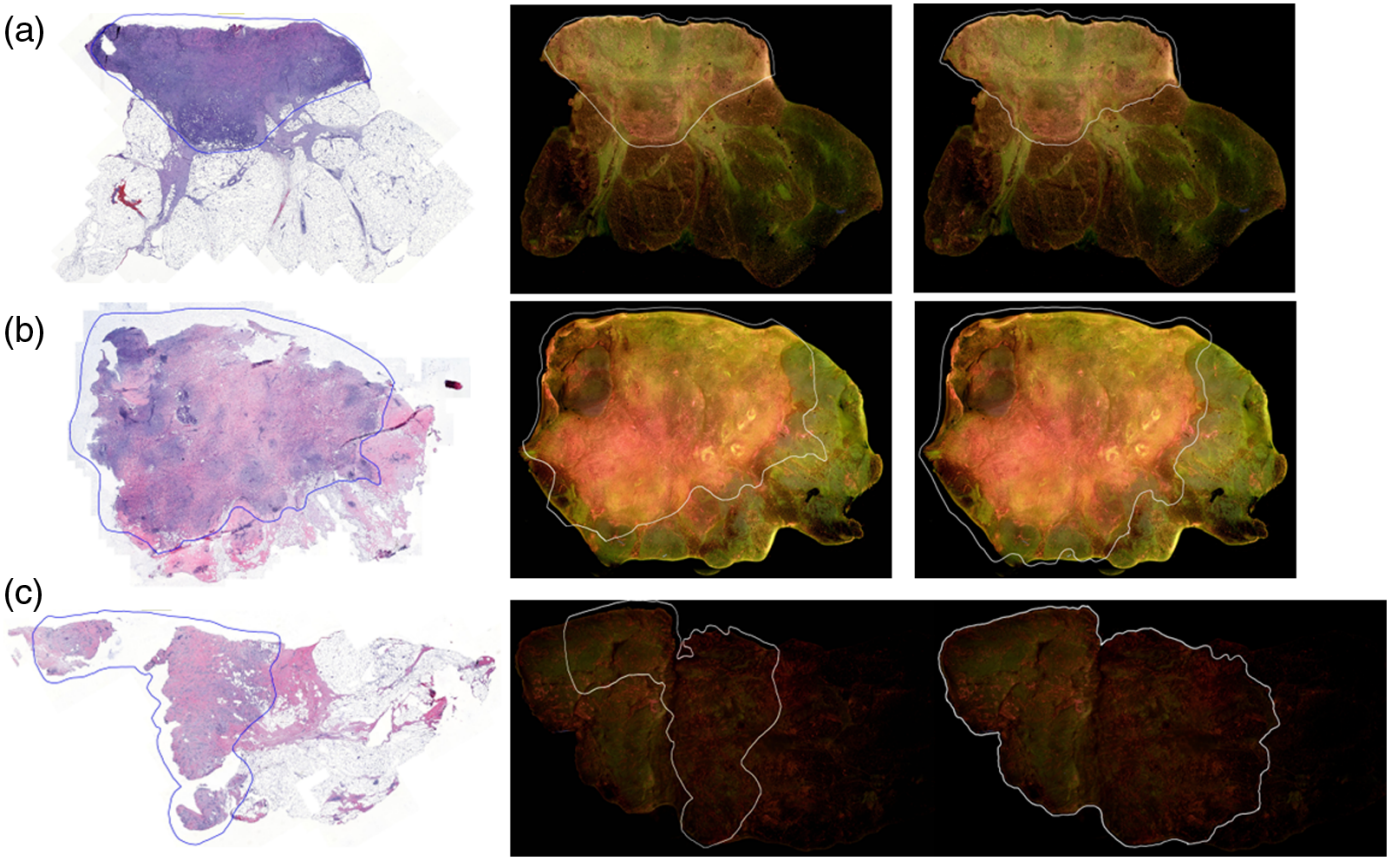
Examples of the pathologist-annotated H&E images (first column) and semi-automatically annotated (second column) and manually annotated (third column) MUSE images of three samples. (a) Sample with excellent performance (Sample #109, IDC, Grade 2, breast tissue) with a DSC of 0.97, CNN scores of 0.97, and Hausdorff distance of 0.06; (b) sample with a medium performance (Sample #160, ILC, Grade 1, breast tissue) with a DSC of 0.90, CNN scores of 0.97, and Hausdorff distance of 0.15; and (c) sample with the worst similarity (Sample #78, IDC, Grade 2, breast tissue) with a DSC of 0.66, CNN 318 scores of 0.88, and Hausdorff distance of 0.19.

**Fig. 7:**
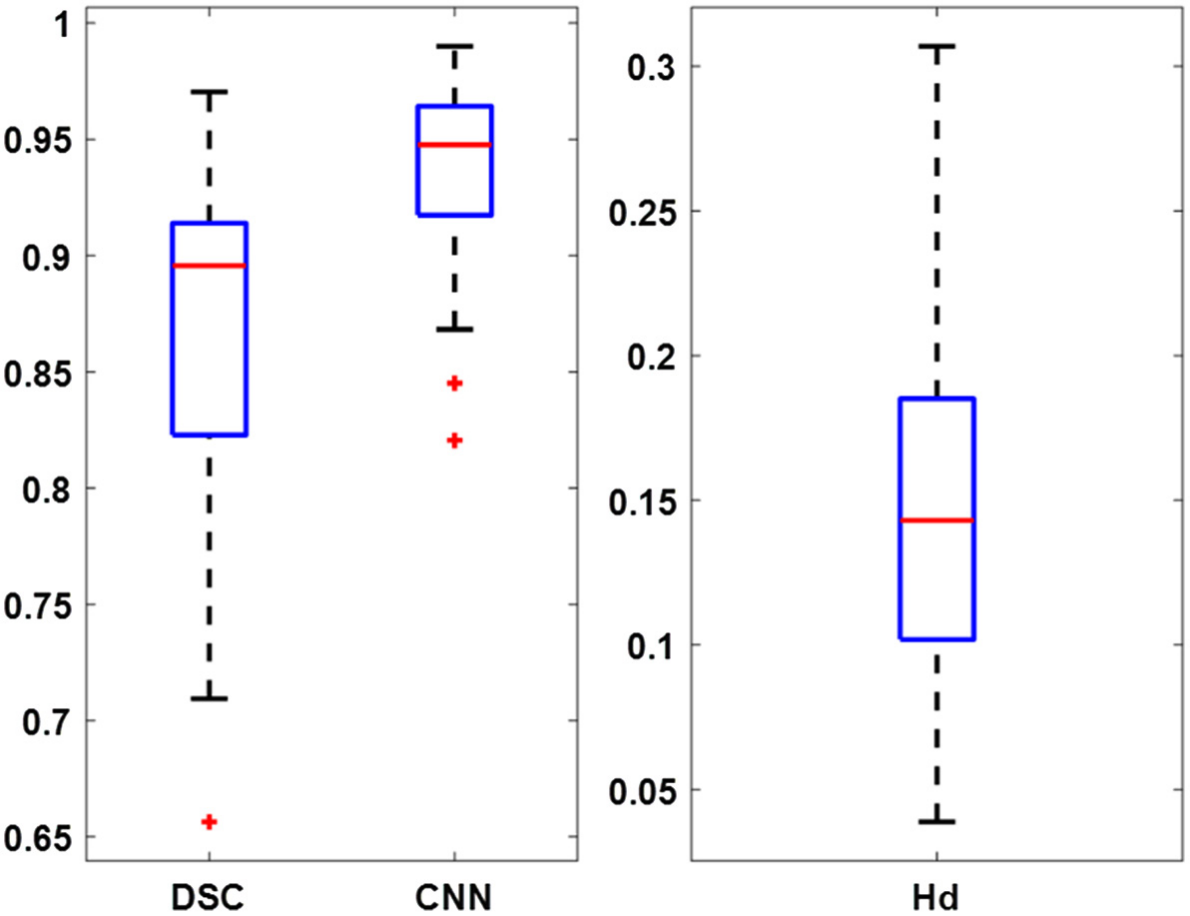
Boxplot of similarity metrics comparing semi-automated and manual annotations on MUSE images across 35 tissue samples. Each box represents the interquartile range (IQR), with the horizontal red line indicating the median. The whiskers extend to the minimum and maximum values within 1.5 times the IQR, and outliers are displayed as individual points.

The semi-automatic annotation approach was utilized to transfer the tumor annotations from the pathologist-annotated H&E images of 35 mixed tissue samples to the corresponding registered MUSE images. The similarity scores were calculated using the DSC, CNN-based feature similarity method, and Hausdorff distance, and the three metrics showed consistent performance among three different researchers. Representative samples with one of the highest, close-to-median score, and lowest similarity score values were chosen to illustrate the excellent, medium, and worst cases, respectively in Fig. 6. The boxplot of similarity metrics for all 35 samples is shown in Fig. 7, and a statistical summary of DSC, CNN-Based Feature Similarity scores, and Hausdorff distances is provided in Table 2.

**Table 2:** Statistical summary of DSC, CNN-based feature similarity, and Hausdorff distance.

	Minimum	Maximum	25^th^ percentile	75^th^ percentile	Median	Outlier(s)
**DSC**	0.656	0.970	0.823	0.914	0.896	0.656
**CNN-based feature similarity**	0.821	0.990	0.917	0.964	0.948	0.821, 0.845
**Hausdorff distance**	0.039	0.307	0.102	0.185	0.143	N/A

The DSC for all samples exhibited an average score of 0.87 across all samples, highlighting substantial consistency in annotation boundaries between the two methods. CNN-based feature similarity, which captures high-level feature correspondence, yielded a higher average score of 0.94. Hausdorff distance, which measures the maximum distance between the annotation lines and is normalized by dividing the image dimension, achieved an average score of 0.15. Furthermore, as indicated in Fig. 7 and Table 2, the DSC ranged from 0.66 to 0.97 with the interquartile range (IQR) spanning from 0.82 (25th percentile) to 0.91 (75th percentile), with a median value of 0.90 and a standard deviation of 0.07, demonstrating a reliable performance across the samples. The CNN-based feature similarity scores ranged from 0.82 to 0.99 with the IQR from 0.92 to 0.96, with a median value of 0.95 and a standard deviation of 0.04, further confirming the consistency of the method. Hausdorff distance varied between 0.04 and 0.31 with the IQR from 0.10 to 0.19, with a median value of 0.15 and a standard deviation of 0.06, indicating a good structural alignment in most cases. These results collectively demonstrate strong agreement between the semi-automated and manual annotations, suggesting that the semi-automated approach performs well in transferring tumor annotations from H&E to MUSE images.

## 4 Discussion

DL-based MUSE imaging has great potential for intraoperative assessment of tumor margins but requires a large number of annotated images for model training and testing. The developed semi-automatic method aimed to transfer annotations accurately and efficiently from pathologist-annotated H&E images to MUSE images, thus facilitating the development of DL-enabled MUSE technology for intraoperative detection of tumor margins. The results indicated a robust overall performance, with excellent median values for all similarity metrics, including DSC, CNN-based feature similarity, and Hausdorff distance, underscoring the reliability of the method. The low standard deviation across these metrics further highlights its consistency across diverse samples.

This semi-automated method represents a novel approach for transferring tumor annotations from histological H&E images to fluorescence images captured by MUSE. To the best of our knowledge, this is the first effort to achieve annotation transfer in this context without relying on segmentation. Previous studies have explored annotation transfer between different imaging modalities, such as between H&E images and images acquired by MSI in a pancreatic cancer mouse model, where image registration was performed on grayscale images, but no specific accuracy metrics were reported.^65^ In addition, annotation transfer has been attempted between TIRF microscopy and confocal microscopy, focusing on patch-level analysis rather than whole-tissue images.^49^ Registration has also been performed between MRI and Matrix-Assisted Laser Desorption/Ionization Imaging Mass Spectrometry (MALDI IMS) ion images in mouse brain models.^66^ These methods are limited by either the type of imaging modality or the scale of analysis, whereas our method benefits from leveraging whole-tissue, high-resolution fluorescence images generated by MUSE, enabling more accurate and contextually relevant annotation transfer. Moreover, this method can be applied to colored images, in contrast to the grayscale images typically used in existing tools such as the TurboReg plugin in Fiji and AutoAligner by Bitplane Scientific.^49^^,^^67^ This improvement allows for the preservation of richer color-based features, which are particularly important in fluorescence imaging.

Although WSI has been widely used in digital pathology, the use of fluorescence images for intraoperative decision-making remains relatively underexplored.^68^ For example, Mojahed et al.^69^ developed DL classification approaches to distinguish cancerous from noncancerous regions in OCT images of breast tissue, with potential application in IMA. Similarly, DL classification has been applied to Fourier transform infrared spectroscopy and multimodal multiphoton microscopy for tumor detection.^70^^,^^71^ Our approach enhances this process by facilitating the registration of different imaging modalities and enabling the direct transfer of annotations, which offers a more efficient and streamlined method for mapping H&E annotations into different imaging modalities. By facilitating rapid, real-time annotation transfer across whole tissue images, our method is poised to significantly improve the accuracy and efficiency in developing intraoperative tumor classification algorithms, providing a valuable tool for real-time surgical decision-making.

This study has several limitations. Although the method demonstrates strong overall performance, its sensitivity to tissue quality and preparation is evident. First, a few samples exhibited lower scores, including an outlier depicted in Fig. 6(c), which are also reflected in the boxplot of DSC in Fig. 7. The lowest DSC was observed in an IDC grade 2 breast cancer case and was likely due to significant tissue loss during the FFPE process rather than a limitation of the annotation transfer method itself. Because the second-order polynomial registration process depended on manually selected paired points from both H&E and MUSE images, its accuracy was compromised in cases of severe tissue loss or deformation. These findings emphasize the need for further enhancements to improve robustness under challenging conditions, such as integrating automated correction mechanisms or developing adaptive strategies to handle substantial tissue loss such as image reconstruction.^72^^–^^74^ In addition, the registration process is not fully automatic due to the inherent variability introduced by the FFPE process, which can introduce randomness in tissue preparation. Improving these aspects will ensure more consistent, reliable, and faster annotation transfer across different image modalities, ultimately enhancing the quality of datasets used for training DL algorithms. This, in turn, will contribute to the advancement of MUSE and other fluorescence imaging technologies for IMA, facilitating more accurate and efficient tumor classification in real-time clinical applications. In addition to these technical considerations, sample-related factors may also influence performance. Although we did not observe substantial differences in the overall score distributions across samples or subtypes, high-grade tumors generally exhibited better similarity scores. We attribute this trend to the greater cell density and degree of tissue solidity of high-grade tumors, which likely undergo less deformation during FFPE processing compared to low-grade tumors. Furthermore, no obvious score differences were observed between breast and lung specimens, suggesting that tissue type may not be a dominant factor in this context. These findings imply that tumor grade, rather than tissue subtypes or origin, may have a larger influence on the performance of the semi-automated method for annotation transfer. Finally, sample diversity and size remain important limitations, and larger, more varied datasets are necessary to fully validate the robustness of the approach.

Although the proposed annotation method is not designed for real-time intraoperative deployment, it plays a foundational role in the development of DL-based tools for intraoperative tumor classification using MUSE and other fluorescence imaging technologies. By automating the transfer of expert annotations from H&E to MUSE images, this method facilitates the creation of large, high-quality datasets that are essential for training and validating DL algorithms. Future work will involve using this annotated dataset to develop and evaluate DL models capable of providing real-time tumor margin classification in the operating room. This annotation pipeline thus serves as a critical step toward enabling clinically viable, AI-enhanced surgical imaging tools.

## 5 Conclusion

The proposed semi-automatic annotation transfer method enables accurate mapping of tumor annotations from pathologist-labeled H&E images to fluorescence images acquired using MUSE. The method demonstrated high similarity with manual annotations across a diverse dataset, with strong performance metrics in Dice similarity, CNN-based feature comparison, and Hausdorff distance. Although the method itself is not used intraoperatively, it supports the development of DL algorithms intended for intraoperative application by streamlining the generation of annotated datasets. Future studies will focus on leveraging these datasets to train real-time classification models and integrate them with MUSE systems to assist in intraoperative margin detection.

## Data Availability

The MATLAB codes used for registration, annotation transfer, and similarity analysis, along with a representative sample dataset, are publicly available on GitHub at https://github.com/tianling019/MUSE-Annotation-Semiautomated-Transfer. Due to GitHub’s 25 MB file-size limitation, only one representative sample has been uploaded. Additional sample data used to obtain the results in this paper can be requested by contacting the authors at bing.yu@marquette.edu.
